# Geographical genetic variability: a factor to consider when assessing clinical implications of *PRDM9*

**DOI:** 10.1002/mgg3.56

**Published:** 2014-01-19

**Authors:** Alexandra Alemany-Schmidt, Maria Navarro-Palou, Adrià Voltes-Cobo, Jordi Rosell, Damià Heine-Suñer, Antònia Picornell, Maria Oliver-Bonet

**Affiliations:** 1 Laboratori de Genòmica de la Salut, Hospital Universitari Son Espases (HUSE)Palma de Mallorca, Spain; 2 Fundació d'Investigació Sanitària de les Illes Balears (FISIB)Palma de Mallorca, Spain; 3 Secció de Genètica, Hospital Universitari Son Espases (HUSE)Palma de Mallorca, Spain; 4 CIBERER Centro de Investigación Biomédica en Red de Enfermedades RarasMadrid, Spain; 5 Laboratori de Genètica, Institut Universitari d'Investigació en Ciències de la Salut i Departament Biologia, Universitat de les Illes Balears (UIB)Palma de Mallorca, Spain

## To the Editor:

Due to its role during meiosis, variations in the *PRDM9* nucleotide sequence have been associated with different pathologies of meiotic origin: infertility (Irie et al. 2009), de novo genomic disorders (Berg et al. 2010; Borel et al. 2012), and childhood leukemogenesis (Hussin et al. 2013) (OMIM accession number: 609760). So far, alleles of the *PRDM9* zinc finger array have been determined in studies of meiotic recombination, primarily in Icelanders (Kong et al. 2010) and in individuals of central European or African descent (Baudat et al. 2010; Berg et al. 2010, 2011; Kong et al. 2010; Borel et al. 2012; Hussin et al. 2013). To determine the variability in the zinc finger array of *PRDM9* in a Spanish control population (*n* = 103) and in parents of patients with a de novo 22q11.2 deletion (*n* = 16), this genetic region was investigated through Sanger sequencing analysis. Frequencies of alleles found in our control and case populations are shown in Table 1. Four new zinc finger coding repeat types and eight new alleles (Table 1) have been identified. Three of the new zinc finger coding repeat types (spm1 referred as s mutant, spm3 as j mutant, and spm4 as d mutant in (Jeffreys et al. 2012)) and two of the new alleles (L39, L44) had been previously described as de novo somatic/germ-line rearranged *PRDM9* mutants that occur in blood/sperm of individuals carrying known alleles in a publication on *PRDM9* instability (Jeffreys et al. 2012). We show that these “blood/sperm mutants” can also be found as a polymorphic variant in our population. Considering this fact and the number (in the order of hundreds) of *PRDM9* “blood/sperm mutants” found by Jeffreys et al. 2012, one can only expect that this gene's population variability is higher than the variability reported up to now.

**Table 1:** *PRDM9* allelic frequencies observed in our population.

	Spanish						
		Number of alleles		Allelic frequency		European	African
Alleles	Structure	Case	Control	Case	Control ± SE	Allelic frequency	Allelic frequency
**A**	**ABCDEEFGHFIJA**	28	**149**	**0.875**	**0.723 ± 0.031**	**0.840** (*P* = 0.004)	**0.500** (*P* = 0.000)
**L24**	**ABCDDECFTPFQJ**		**11**		**0.053 ± 0.016**	**0.020** (*P* = 0.065)	**0** (*P* = 0.004)
L20	ABCDDECFGKFQJ		7		0.034** ± **0.013	0.040	0
B	ABCDDCCFGHFIJ	2	6	0.063	0.029** ± **0.012	0.020	0.047
**L26**	**ABCDDECFGHFQJ**		**6**		**0.029 ± 0.012**	**0.005** (*P* = 0.057)	**0** (*P* = 0.036)
L19	ABCDDCCFKHLHQIJ		5		0.024** ± **0.011	0	0.014
D	ABCDDECFKGHFIJ		4		0.019** ± **0.001	0.010	0
L32	ABCZDECFGHFIJ		3		0.015** ± **0.008	0	0
C	ABCDDCCFKHLHIJ		3		0.015** ± **0.008	0.010	0.128
L9	ABCDDECFGPFQJ		2		0.010** ± **0.007	0.010	0
L39^1^	ABCFGHFIJ		2		0.010** ± **0.007	0	0
L1	ABCDGHFIJ		1		0.005** ± **0.005	0.003	0
E	ABCDHFIJ		1		0.005** ± **0.005	0.019	0
L15	ABCDDCCFKHLHI	1		0.031	0	0	0.020
L40^1^	ABCDDECFQJ		1		0.005** ± **0.005	0	0
L41^1^	ABCDspm3DDECFGHFIJ		1		0.005** ± **0.005	0	0
L42^1^	ABCDDECFGspm1FIJ		1		0.005** ± **0.005	0	0
L43^1^	ABCDDECFGHFHIJ		1		0.005** ± **0.005	0	0
L44^1^	ABCDDECFGHFI		1		0.005** ± **0.005	0	0
L45^1^	Aspm2CDDECZGHFIJ		1		0.005** ± **0.005	0	0
L46^1^	ABCDDECspm4CHFIJ	1		0.031	0	0	0
Others						0.023	0.311
N		32	206	1.0000	1.0000	206	148

Diversity indices				Spanish (control)		European	African
Number of different alleles				19		16	19
Observed heterozygosity				0.4563		–	–
Gene diversity				0.4717** ± **0.4568		0.2927** ± **0.3426	0.7320** ± **0.6066
Pair-wise comparisons^2^							
Spanish (control)				–		0.01895	0.06398
Europe				**0.00901 ± 0.0091**		**–**	0.15428
Africa				**0.00000 ± 0.0000**		**0.00000 ± 0.0000**	–

Comparison of data obtained in control population with European and African data (Berg et al. 2011). Allele A (GenBank reference sequence: GU216222.1) in this Spanish population presented an intermediate position between African and central European populations, although the overall distribution of *PRDM9* alleles was closer to Europeans than to Africans. The African influence in this Spanish population, as shown by the presence of an African allele (L19) and the A allelic frequency, is in accordance with other genetic studies of European Mediterranean populations. Bolded alleles have higher interpopulation differences (regarding control population).

1 New alleles found in this study. New alleles have been submitted to GenBank (accession numbers KF475787-KF475794).

2 Pair-wise comparisons: above diagonal – Reynold's distances; below diagonal – Fst *P* values (significant values are bolded).SE, standard error.

When analyzing the possible clinical implications of *PRDM9* as a risk factor for genomic instability, Berg et al. (2010) obtained results that suggested that rare allelic forms of this gene confer a protective effect against rearrangements in three highly unstable minisatellites and de novo CMT1A and HNPP rearrangements. Later, Borel et al. (2012)) showed that the majority of the de novo microdeletions they studied were not dependent on non–A alleles. In accordance to all these previous results, we also observe a higher frequency of the A allele in our case population (87.5%) than in our control population (72.33%) (Table 1). The sample size of our case group, transmitting parents of a de novo 22q11.2 deletion, however, is too small to achieve statistical significance.

Also regarding *PRDM9* clinical relevance, it has been suggested that differences in *PRDM9* allelic frequencies between populations correlates with differences in the susceptibility to de novo rearrangements (Berg et al. 2010). If true, this hypothesis could be extended to other diseases in which an implication of *PRDM9* is assumed. For example, a recent study (Hussin et al. 2013) associates rare allelic forms of *PRDM9* with childhood leukemogenesis. The authors observed a statistically significant higher frequency of both non–A alleles and k-finger alleles (alleles containing a k zinc finger coding repeat type) in parents of children with acute lymphoblastic leukemia (ALL) (32.7% and 13.5%, respectively) compared to controls (15.1% and 3.3%, respectively). In our control population, non–A alleles and k-finger alleles (C, D, L19, and L20) were observed at an allelic frequency of 27.67% and 9.2%, respectively. It would be interesting to replicate this study in a population with a higher incidence of k-finger alleles, such as the one described in this article, in order to confirm the implication of these alleles in the generation of ALL.

Given the differences observed among only three different populations (Table 1) and considering the many “blood/sperm mutants” found by Jeffreys et al. (2012), we believe that future studies aiming to increase understanding of the role of *PRDM9* alleles in determining human susceptibility to meiotic disorders or other diseases would benefit from having a broader picture of *PRDM9* variability in human populations.

## Supporting Information

Data S1 Data about control and case population.

Data S2 Statistics.
