# Supplementary material for: Geographical genetic variability: a factor to consider when assessing clinical implications of PRDM9
Source: Mol Genet Genomic Med. 2014 Jan 19;2(2):201–3. doi: 10.1002/mgg3.56 (PMC3960062; doi:10.1002/mgg3.56)
Supplement: Data S1 — Data about control and case population. [file mgg30002-0201-sd1.doc]

Supporting information 1

Data about control and case population

All DNAs of patients with 22q11.2 deletions (which are the cause for DiGeorge and Velocardiofacial syndromes) (1) have been characterized by either MLPA (MRC-Holland, salsa P250) or SNP-array (Illumina, Infinium 660W) and found to correspond to the 3 Mb recurrent deletions which are the most frequent and are generated by non-allelic homologous recombination (NAHR) (2, 3). Microsatellite analysis using an in-lab panel (unpublished) determined that deletions were de novo, identified the transmitting parent (in which the deletion occurred) and non-transmitting parent, and confirmed that markers flanking the deletion had recombined and that thus, the deletion had been generated by NAHR.

The control group consisted of a heterogeneous population of unrelated Spanish individuals currently living in Mallorca (Balearic Islands), comprising both females and males, with ages ranging between 21 and 86. Geographic origin of all individuals was assigned on the basis of both paternal and maternal grandparents' place of birth.

1. McDermid HE, Morrow BE. Genomic disorders on 22q11. Am J Hum Genet 2002: 70: 1077-1088.

2. Edelmann L, Pandita RK, Spiteri E et al. A common molecular basis for rearrangement disorders on chromosome 22q11. Hum Mol Genet 1999: 8: 1157-1167.

3. Emanuel BS. Molecular mechanisms and diagnosis of chromosome 22q11.2 rearrangements. Dev Disabil Res Rev 2008: 14: 11-18.
