# Supplementary material for: Geographical genetic variability: a factor to consider when assessing clinical implications of PRDM9
Source: Mol Genet Genomic Med. 2014 Jan 19;2(2):201–3. doi: 10.1002/mgg3.56 (PMC3960062; doi:10.1002/mgg3.56)
Supplement: Data S2 — Statistics. [file mgg30002-0201-sd2.doc]

Supporting information 2

Statistics

Allelic frequencies, Nei’s gene diversity index (1), heterozygosity and Hardy-Weinberg Equilibrium (HWE) were estimated using ARLEQUIN software, version 3.11 (2). Pairwise Fst and associated probability values, as well as Reynolds’ genetic distances were estimated with the methods of Slatkin (3) and tested for signiﬁcance by permutation (ARLEQUIN v 3.11), in order to compare the control population with literature populations (4, 5). The comparison of the frequencies of A, L24 and L26 alleles between populations was carried out by means of the Fisher’s exact test in a contingency table (6).

1. Nei M. Molecular Evolutionary Genetics. Columbia University Press, New York, 1987.

2. Excoffier L, Laval G, Schneider S. Arlequin (version 3.0): an integrated software package for population genetics data analysis. Evol Bioinform Online 2005: 1: 47-50.

3. Slatkin M. A measure of population subdivision based on microsatellite allele frequencies. Genetics 1995: 139: 457-462.

4. Berg IL, Neumann R, Lam KW et al. PRDM9 variation strongly influences recombination hot-spot activity and meiotic instability in humans. Nat Genet 2010: 42: 859-863.

5. Berg IL, Neumann R, Sarbajna S et al. Variants of the protein PRDM9 differentially regulate a set of human meiotic recombination hotspots highly active in African populations. Proc Natl Acad Sci U S A 2011: 108: 12378-12383.

6. Sokal RR, Rohlf FJ. The Principles and Practice of Statistics in Biological Research. Freeman, New York, 1994.
